# SPEED (Supporting Psychiatry Experience and Education in District-Hospitals), a Pilot Program for Foundation Doctors

**DOI:** 10.1192/bjo.2024.294

**Published:** 2024-08-01

**Authors:** Sam Fraser, Sophie Clark

**Affiliations:** NHS Ayrshire and Arran, Ayr, United Kingdom

## Abstract

**Aims:**

This pilot program aimed to enhance the psychiatry experience for foundation doctors (FYs) working at Ayr Hospital by identifying perceived areas where psychiatric support would benefit training, development or education. Subsequently strategies were aimed to be implemented by the psychiatry liaison service to enrich FYs' experience during their medical and surgical rotations. Feedback was aimed to be obtained to determine if the program would have value to other district hospitals and grades of junior doctors.

**Methods:**

Unstructured interviews with 4 FYs were conducted in October 2023 to explore the current experience of psychiatry in medical or surgical placements at Ayr Hospital. Identified themes included barriers to completing supervised learning events (SLEs) for mental health cases (a requirement of the 2021 Foundation Curriculum), limited exposure to psychiatry teaching opportunities, and obstacles to pursuing development of interest in psychiatry (such as time to shadow psychiatry, or discuss career prospects in psychiatry).

A pilot program was initiated in November 2023 to improve the experience and education of psychiatry for FYs. This involved:

- Providing dedicated time on wards for FYs to complete SLEs with a member of the liaison service.
- Providing time for FYs to shadow the role of liaison psychiatry.
- Providing additional teaching tutorials, focused on topics chosen by FYs.
- Providing the opportunity to discuss and develop interest in psychiatry.

A survey to obtain both quantitative and qualitative feedback was sent to each FY that engaged in the program.

**Results:**

17 FYs engaged in the pilot program, with 13 providing feedback. All respondents felt the program increased their knowledge and confidence in approaching cases with a psychiatry element. They also all found the experience positive and a productive use of time. All deemed the program would be useful for other foundation trainees in medical hospitals. Free text feedback highlighted the program's value in facilitating case discussions, removing obstacles in completing mental health SLEs, providing useful relevant tutorials and providing opportunity to discuss further interest of psychiatry.

**Conclusion:**

The pilot program successfully achieved its aim to improve FYs' experience of psychiatry. Although not measured in the survey, the program also appeared to foster positive relationships between the liaison service and junior medical staff. It also helped identify new appropriate referrals for the liaison service. An expansion of the program is planned to other district hospitals in Ayrshire and Arran, including consideration of expanding the participation to wider members of the junior doctor cohort.

